# Branching Vine Robots for Unmapped Environments

**DOI:** 10.3389/frobt.2022.838913

**Published:** 2022-03-24

**Authors:** Paul E. Glick, Iman Adibnazari, Dylan Drotman, Donald Ruffatto III, Michael T. Tolley

**Affiliations:** ^1^ Department of Mechanical and Aerospace Engineering, University of California, San Diego, San Diego, CA, United States; ^2^ NASA Jet Propulsion Laboratory (JPL), La Cañada Flintridge, CA, United States

**Keywords:** soft robotics, vine robots, optimization, design, eversion

## Abstract

While exploring complex unmapped spaces is a persistent challenge for robots, plants are able to reliably accomplish this task. In this work we develop branching robots that deploy through an eversion process that mimics key features of plant growth (i.e., apical extension, branching). We show that by optimizing the design of these robots, we can successfully traverse complex terrain even in unseen instances of an environment. By simulating robot growth through a set of known training maps and evaluating performance with a reward heuristic specific to the intended application (i.e., exploration, anchoring), we optimized robot designs with a particle swarm algorithm. We show these optimization efforts transfer from training on known maps to performance on unseen maps in the same type of environment, and that the resulting designs are specialized to the environment used in training. Furthermore, we fabricated several optimized branching everting robot designs and demonstrated key aspects of their performance in hardware. Our branching designs replicated three properties found in nature: anchoring, coverage, and reachability. The branching designs were able to reach 25% more of a given space than non-branching robots, improved anchoring forces by 12.55×, and were able to hold greater than 100× their own mass (i.e., a device weighing 5 g held 575 g). We also demonstrated anchoring with a robot that held a load of over 66.7 N at an internal pressure of 50 kPa. These results show the promise of using branching vine robots for traversing complex and unmapped terrain.

## 1 Introduction

Robotic exploration of unfamiliar environments is a fundamental problem made more difficult when the terrain impedes the use of traditional wheeled, legged, or flying robots. Relevant examples include collapsed buildings for search-and-rescue applications (Davids, 2002), coral reef cavities for ecological monitoring (Luong et al., 2019), cave networks for mining or rescue tasks (Parcheta et al., 2016; Ebadi et al., 2020), shipwrecks for archaeological surveys (Ballard et al., 2002), and the human body during medical interventions (Berthet-Rayne et al., 2021; Li et al., 2021). Many of these environments are characterized by tight spaces, further complicating the task of exploration by precluding the use of onboard energy storage and motivating tethered designs. Challenges of data transmission over long distances and through certain media further push systems towards the use of tethers (Otsu et al., 2020). However, tethers limit mobility and can generate significant friction due to the capstan effect (Blumenschein et al., 2017). Tethers can significantly limit the exploration of confined spaces since the force from this friction grows exponentially with the total change in angle along the tether (which, in general, is a function of the geometric complexity of the environment). Another challenge of navigating these environments is the non-planar nature of many of the spaces. Wheeled or legged robots that are constrained by gravity may face impassable obstacles (Krotkov et al., 2017). Flight offers a potential solution, but is energy intensive and generally only works in relatively large spaces (Sabet et al., 2019). Finally, the exploration of these constrained and unmapped environments often leads to physical interactions between the robot and the environment that can result in damage to the robot, the environment, or both.

**FIGURE 1:**
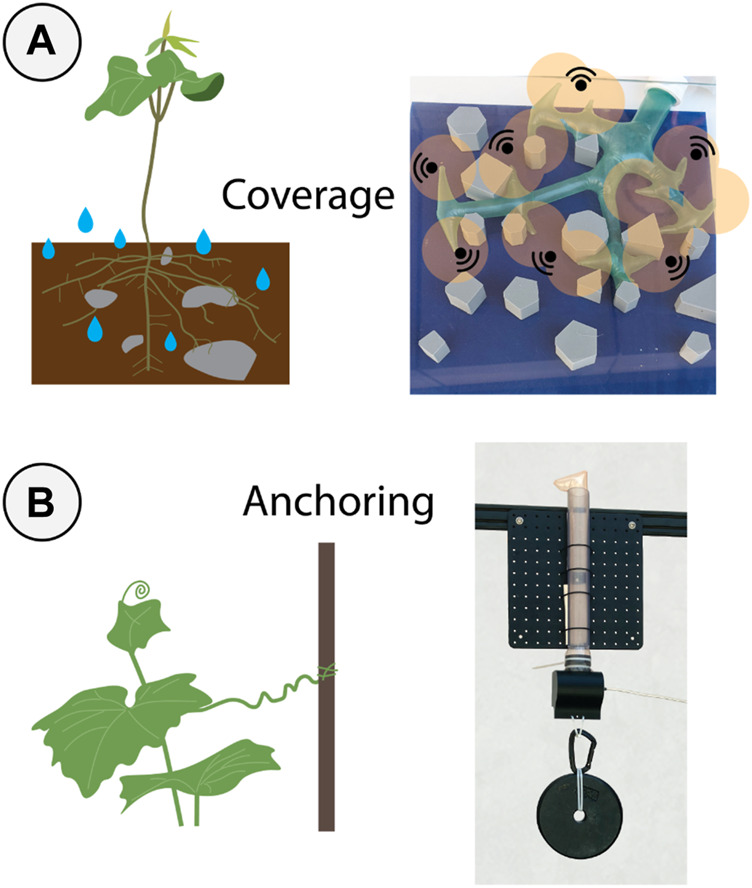
We developed branching vine robots for navigating and anchoring in unexplored spaces. **(A)** Distributing branches across a larger space allows for greater simultaneous coverage for water collection in nature (left) or sensor placement for robotics. Additionally, using many different routes for growth improves the reachability of the system in case one path becomes stuck in an obstacle such as a rock or wall (right). **(B)** Plants are able to anchor to their surroundings for structural reinforcement (left). We demonstrated anchoring with a single branch growing through a confined space, then supporting a payload of 66.7 N (15 lbs) at an internal pressure of 50 kPa (right).

Recently, there has been great interest in soft growing robots that address many of the challenges that complicate the exploration of these environments (Coad et al., 2019). Robotic systems capable of growth have been realized most notably through the pressure driven eversion of flexible tubes (called *vine robots* or *eversion robots*) (Hawkes et al., 2017), extension of concentric tubes (Morimoto and Okamura, 2016), or the additive extrusion of a thermoplastic (Sadeghi et al., 2017). By growing the robot at the distal tip, these growth processes remove the relative motion between the robot body and environment, which can greatly reduce friction (Hawkes et al., 2017). In particular, vine robots are able to leverage their compliance to conform to complex pathways without active steering. With reduced friction, vine robots can penetrate further into confined spaces enabling the ability to deploy to long lengths (1–10 m) without concerns regarding power and data transmission (Hawkes et al., 2017). Another key advantage of growing robots is the self-supporting nature of their deployed structures (Wang et al., 2020; Blumenschein et al., 2021). Since the grown structure supports new material deployed at the tip of the robot, the new growth can be directed freely in 3-D space within limits defined by steering and payload capabilities (Greer et al., 2019). This 3-D mobility gives growing robots the ability to follow complex pathways and avoid obstacles. Finally, soft growing robots are not hindered and are even aided by rich interactions with the environment (Greer et al., 2020). Structural compliance seen in soft robots allows for adaptation to irregular features that would otherwise hinder rigid systems. Due to their compliance, both the robot and environment are unlikely to be harmed by interaction (Polygerinos et al., 2017; Haggerty et al., 2019). Furthermore, these environmental interactions are helpful for soft growing robots, aiding with navigation (Greer et al., 2020), and helping to support the deployed structure and increase payload capacity (Luong et al., 2019). By contrast, most mobile robots must conduct path planning to carefully avoid obstacles (Greer et al., 2020). A direct analog can be found in nature with root and vine structures that would not be self-supporting without external reinforcement (Wooten and Walker, 2018) (see Figure 1). For these reasons, pressure-driven soft growing robots hold great promise for the exploration of narrow and complex passageways. However, this promise is not yet fully realized for passageways that have not previously been mapped.

Due to the challenge of supporting a large payload with a soft growing structure, approaches for steering eversion robots have bifurcated into: active steering (Sadeghi et al., 2017; Greer et al., 2019; Satake et al., 2020) and obstacle-aided navigation (Luong et al., 2019; Greer et al., 2020; Selvaggio et al., 2020). Active steering approaches face challenges in unexplored spaces due to the spatial constraints of the environment and the difficulty associated with carrying mass at the tip of the robot for sensors or actuators. Vine robots may be gentle enough to adapt to their environment without risk of damage, but the trade-off for this compliance is a limited ability to support payloads. The maximum payload that these robots can support depends on the length deployed, getting smaller as the robot grows (Luong et al., 2019). Additionally, in small spaces, a bulky payload at the tip can limit mobility and restrict the robot to only growing through orifices larger than the payload diameter (Jeong et al., 2020). For these reasons, everting robots are most capable when unencumbered by payloads at the tip (Hawkes et al., 2017).

This behavior drives designs towards smaller and simpler payloads which conflicts with the sensing and actuation hardware required to map and navigate unknown environments. A solution may be found through open-loop growth, reliant on a rich use of obstacle-interactions to help guide the robot. Instead of active steering, designers can leverage the compliance of vine robots to conform to preexisting paths in the environment. The promise of this approach is compelling, allowing the robot to deploy without much mass its tip. For environments that have been mapped in advance, this obstacle-aided navigation can be used to reach a desired location reliably and easily without any active control (Greer et al., 2020). However, in the absence of prior knowledge of the environment, a straight robot body is limited by the condensation of many robot paths into a single vertex of an obstacle, as explained by Greer et al. (2020). This phenomenon of trajectory condensation means that open-loop growth requires some predefined bends or structure in the robot to improve the reachability in the workspace. Yet specific placement of these bends requires prior knowledge, precluding the use of this approach in unmapped spaces. Researchers are developing planning and control algorithms using a hybrid of both obstacle-interactions and active steering (Ataka et al., 2020), but this does not address the fundamental limitations of each in a confined and unexplored space.

To realize the potential of vine robots for unmapped spaces, we propose the use of branching vine robots. We investigate the question of how to optimize these structures for operation in unmapped spaces. To test this, we developed a customizable framework for optimizing the design of branching structures by leveraging a set of known maps from a target environment. We simulated the deployment of branching structures on those known maps. We paired application-specific reward heuristics with population-based optimization methods to find high-performing designs to evaluate on unseen maps from the same training environment. We used both anchoring and sensor-coverage as two different example tasks for our optimization process, and tested some of the optimized branching designs in hardware. We also fabricate branching vine robots capable of repeated deployment. Finally, we demonstrate a self-deploying anchor for heavy payloads.

## 2 Materials and Methods

Vine robots are soft structures capable of growth driven by internal pressure. Their intrinsic compliance allows for obstacle aided navigation in cluttered spaces (Greer et al., 2020). Branching vine robots, a type of growing robot that are characterized by having several apices each of which is capable of deploying new material, include a hierarchical growth pattern that significantly expands the design space. We studied the optimization of branching designs in response to this expansion of the design space.

### 2.1 Optimization

**FIGURE 2:**
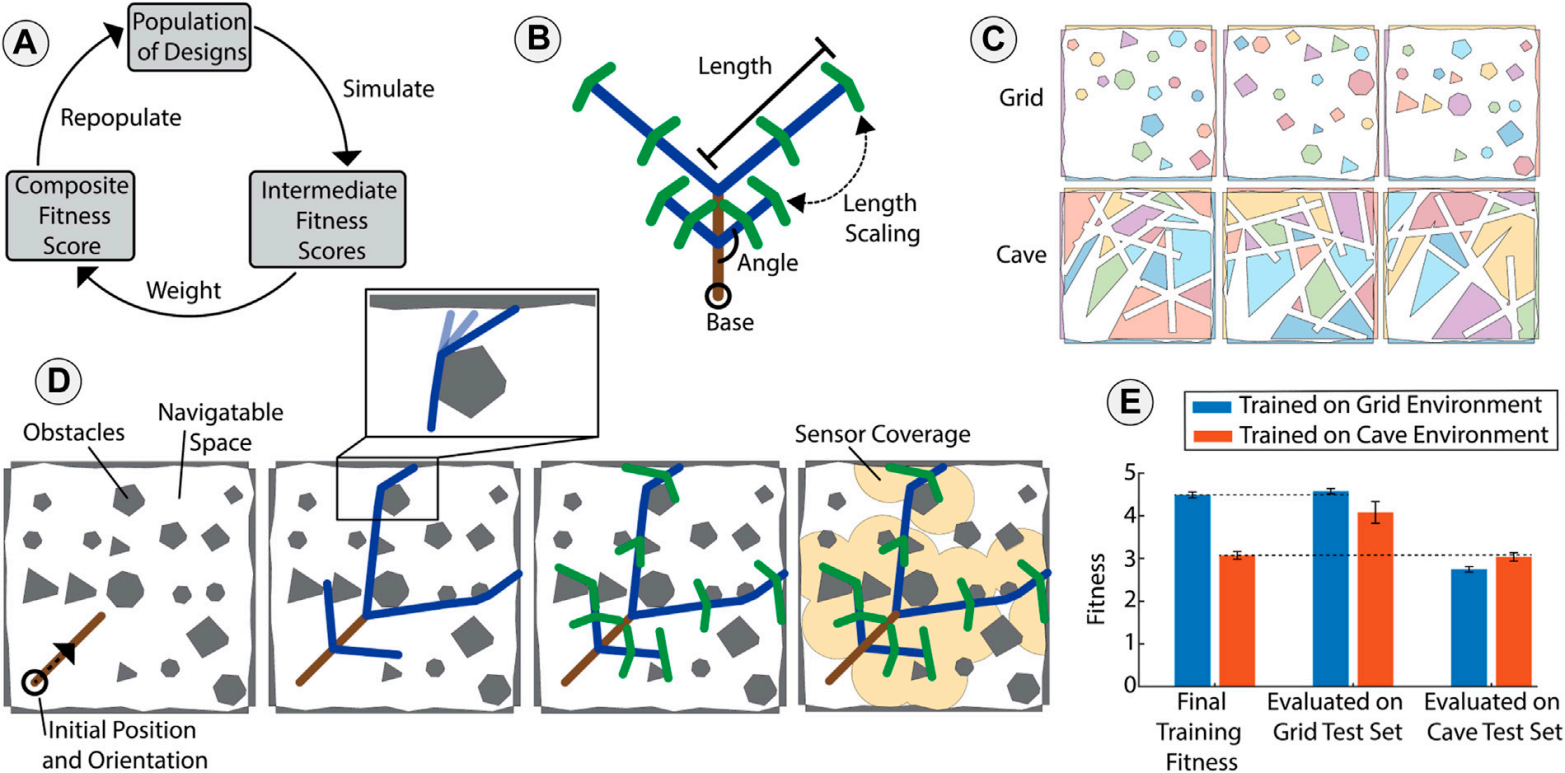
Optimization of branching vine robots through simulation. **(A)** We used a particle swarm algorithm to guide the optimization of the robot design. Within a generation, each design was evaluated on several maps by simulating open-loop growth. We assigned a fitness score for each evaluation using a task-specific heuristic. These fitness scores were then combined using weighted averages into a single composite fitness score, and the designs were then repopulated based on the composite fitness scores. **(B)** Representative parameterized robot design. Each level of the hierarchy is represented with a different color. Within a level, branch lengths, angles, and a growth scalar determine the morphology. **(C)** Examples of simulated maps from the simulated *Grid* and *Cave* environments. Each obstacle is represented by a distinct color. **(D)** Visualization of a representative output from the simulator, where the final position of the branching robot is predicted based on initial orientation, the map, and the design of the robot. The simulator simulates the deployment of each level of the branching structure using a recursive open-loop growth algorithm. After simulating growth, we assigned the fitness score based on the chosen task (in this case sensor coverage). Inset: buckling about a distal obstacle during collision. **(E)** Comparison of optimization results for designs trained in different environments. The performance of optimized designs transferred well from training environments to unseen test maps (dashed lines). Additionally, each design specialized to its training environment.

To optimize the design of these structures we used heuristic rewards calculated from simulation. (Figure 2A). To provide a reward estimate for the optimization process, we developed a simulator that predicted the final position that an eversion robot reaches through open-loop growth given 1) a known map, 2) an initial state (position and angle), and 3) a parameterized design morphology. The reward was based on an application-specific heuristic calculated from the final state of the robot. Tuning this reward prioritized different spatial properties such as exploration and area-coverage or kinematic properties such as anchoring forces. We considered two different categories of spaces for the optimization, environments (e.g., coral reefs, caves) and maps (i.e., an individual instance of a particular environment with a single set of obstacle shapes and locations). Since this process still required known maps to calculate expected rewards, we leveraged a set of known archetypal maps from the same environment as the unmapped target application location. Through this process we were able to optimize for an environment rather than for any one specific map, allowing us to design for and test performance on unseen maps. A necessary property for results to transfer from the known to unknown instances of a specific environment is that there should be some overlapping similarities within the environment. While further work is needed to validate this for any particular real-world environment, we enforce this property through the use of computer generated environments generated through a randomized but controlled process. We detail this optimization framework in the following section.

### 2.2 Simulation Framework

To predict the growth process of a branching eversion robot in open loop, we simulated the deployment of branches recursively, letting us adapt a set of well-established algorithms. Kinematics and buckling in inflatable beams are well studied (Hammond et al., 2017; Haggerty et al., 2019). Greer et al. consolidated these into a set of algorithms that predict the final position of an eversion robot given the initial state, map, and body-length of the robot (Greer et al., 2020). This work has since been refined and the simulation of soft growing robots has been developing rapidly (Selvaggio et al., 2020; Jitosho et al., 2021). These algorithms predict how a vine robot will deflect as it comes into contact with obstacles, relying on the contact mechanics of inflatable beams (Blumenschein et al., 2017) and assuming the robot will always buckle about a distal environmental contact (Hammond et al., 2017). The specific contact point from which the robot will deflect is determined by the relative orientation of the robot and the obstacle. The robot is assumed to grow tangentially along an obstacle while in contact (Greer et al., 2020). These algorithms were validated experimentally and can be adapted to include analysis of confidence or the use of pre-formed bending designs (Greer et al., 2020; Jitosho et al., 2021).

We built on this prior work to include branching by treating branched growth as a recursive problem (Figure 2D). Branching has been previously introduced for reconfigurable antennas (Hawkes et al., 2017; Blumenschein et al., 2018) and has also been seen in continuum robots (Lastinger et al., 2019), but has not yet been considered for mobility or obstacle interaction in growing vine robots. We assumed that each hierarchical level of branching deployed sequentially and that the forces resulting from object interactions in one branch did not impact the position of the remaining structure. This assumption relies on having enough external interactions with the environment to constrain any deployed material. To allow for morphological changes during optimization, we parameterized the design space. The number of design variables depended on the morphological complexity of the design. The design parameterization included the number of levels in the branching hierarchy, *n*. The other parameters were the lengths of the branches in a level, *BL*_*i*_, starting angle of new branches in a level, *θ*_*i*_, number of new branches in a level, *SPL*_*i*_, and a length growth scalar that added variation to the lengths of branches within a level, *LGF*_*i*_ (Figure 2B). These included a separate instance for each level of the hierarchy, denoted by *i*. The parameters along with their respective optimization limits are presented in Table 1. Along with a parameterized design approach and the algorithms to predict growth, optimization also required a set of maps and a heuristic reward function. Since each fitness evaluation depended on the specific map and starting orientation, each evaluation only represented an approximation of the true fitness for a particular design across a type of environment. Therefore, we evaluated fitness for each design on several map instances using task-specific heuristics. We then weighted these fitness scores into a single composite fitness for each design. This weighting allowed the user to tune the optimization process given the variability in a particular environment. This weighting was chosen to prioritize average performance, a “greedier” peak performance, or the minimization of variability.

**TABLE 1:** Parameters and their respective bounds for the optimization.

Parameter	Lower and upper bounds
*n*	2 to 5
*BL*	10 to 205
*θ*	20 to 160
*SPL*	2 to 4
*LGF*	10 to 160

#### 2.2.1 Example Applications

To realize desirable optimization results, the maps and reward function used to guide the design search were tailored to the intended application. We wrote our simulator to accept 2D maps containing a randomized number and shape of obstacles. Obstacles in the map were closed polygons defined by a set of ordered *x* and *y* coordinate pairs. To optimize for exploration of unmapped spaces in a known target environment, we used a set of known maps from the same environment in the optimization. To control the randomness between map instances within an environment, we created map-generating algorithms which each produced randomized polygons following a set of environment-specific rules (see Supplementary Information S.I.-A). This is inspired by real-world environments where even if each instance is unique, the same geological or biological processes drive the creation of an environment. We controlled properties such as obstacle shape, density, and convexity. Such properties are not guaranteed to transfer across real world environments, but controlling these parameters allowed us to generate randomized environments with inherent similarities. In our experiments we used two computer-generated types of environments—referred to as a *Grid* environment and a *Cave* environment (Figure 2C). The grid maps consisted of a high number of convex polygons, and the cave maps contained a series of interconnected closed pathways. Details on how we generated these environments are presented in the Supplementary Information S.I.-A. While map sets were selected from one of these environment types, we defined the fitness functions as an application-specific user-defined heuristic. Since the simulation and model predict the final state given input parameters, a fitness function was required to define what constituted a successful design. The following subsections detail example tasks and the fitness metrics used to evaluate designs. We used two different biologically inspired applications as examples in this work. The fitness functions used below can be found in Supplementary Information S.I.-C.

### 2.3 Sensor Coverage

In this example task, the objective was to distribute sensors throughout a cluttered space to maximize coverage with the simplest branching robot design. To calculate a reward, we added a post-processing step to the simulator. At the end of growth, each unique branch deployed a sensor with fixed circular area of coverage at its tip. Since the goal was to maximize area coverage, we only considered unique area as productive. Therefore, we calculated the reward as the union of the area covered by sensors. To balance design simplicity with area coverage we added a cost that depended on several variables. The first consideration for the cost was the efficiency of the sensor coverage. In other words, if the covered areas were not overlapping, each sensor was providing productive information. We penalized both the number of total sensors and the total length of the robot design, where each was scaled by the efficiency of the covered area (how much of the sensed area overlapped). Additionally, we added a small penalty based purely on the total length of the design to minimize the design in the case that two designs had similar efficiency. This is because a simpler (i.e., shorter total length) design requires less mass and also would use energy to deploy. Finally, we normalized each of these four values (union of the sensor area, number of branches scaled by efficiency, length of design scaled by efficiency, and raw length) to fall in the range [0,1] and then weighted them to balance coverage and simplicity (equations in Supplementary Information S.I.-C).

### 2.4 Anchoring

Analogous to root growth in plants, the goal in this task was to distribute forces across the branched structure to anchor into the environment. Even with low internal pressures, we assumed anchoring forces would become appreciable due to two synergistic effects: First, we assumed each branch would act as an inflatable anchor, capable of jamming in narrow or constrained spaces (Eq. 1) where *F*_*tip*_, *P*, *A*_*c*_, and *μ* represent the anchoring force of the pouch, internal pressure, area in contact, and coefficient of friction respectively (Glick et al., 2020). Second, we assumed that as the robot wrapped around or grew past obstacles, the capstan effect produced an exponential increase in anchoring force (Eq. 2) with *F*_*anchor*_ as the anchoring force of the branch and gamma as the total change in angle along the path of the branch (Blumenschein et al., 2017; Luong et al., 2019). We assumed that this capstan effect would amplify the frictional increase from jamming in narrow spaces.

$$F_{tip}=2\cdot P\cdot A_{c}\cdot \mu$$ (1)

$$F_{anchor}=F_{tip}\cdot e^{\gamma \cdot \mu }$$ (2)

For our fitness score, we used a nondimensional scaling of the anchoring force. The anchoring force for each branch was measured based on the interaction with obstacles which determines the jamming force and capstan friction. We assumed branches were narrow and did not provide any jamming on their own, and instead relied on a pouch anchor, larger inflatable section to provide known contact area, at the tip of each branch to provide the base anchoring force for that branch (Glick et al., 2020). This allowed us to calculate anchoring forces for the branched robot based on the total angle change of each branch, where the total anchoring force is the sum of the anchoring force of each branch. This modelling and design approach allowed us to model, predict, and verify anchoring forces easily. In reality, if branches are not narrow and are at the same scale as the space between obstacles, anchoring forces would be distributed across the entire length of the contact area. This would likely increase total anchoring forces and improve resilience in the case of any slipping, but presents a much more difficult case to model and verify. While in practice, that magnitude of the anchoring force (measured in N) would depend on the internal pressure of the robot, the size of the pouch at the tip, and the friction of the material of the anchors, we assumed that these properties would scale the magnitude of the anchoring force equally for all branching morphologies, thus we ignored them during optimization. Therefore, for optimization and hardware validation we treated anchoring as a non-dimensional scalar, measured as the anchoring force of the entire deployed branching robot divided by the anchoring force of single pouch without any capstan effect. For this task, we used a simplified design space to improve accuracy of the model predictions, so we did not add a penalty to the fitness function for the complexity of the design.

#### 2.4.1 Search Methods

We tested multiple gradient-free optimization techniques that enabled the optimization of branching eversion robots for operation in unmapped environments. In Supplementary Information S.I.-C, we show both a customized evolutionary algorithm and a commercially available particle swarm algorithm (Matlab *particleswarm* Solver) that both yielded similar performance (Panda and Padhy, 2008). For the results in this paper, we used the particle swarm algorithm since it was slightly faster. The lack of an explicit and differentiable objective function motivated our use of gradient-free search methods. The challenge of selecting a search method was complicated by the fact that the fitness landscape was heavily dependent on the task-specific fitness function and the set of maps used in the training phase. Furthermore, since there was symmetry and redundancy in the design space, some dissimilar designs performed equivalently. Though there is no guarantee that a single global optimum existed, we did not test local search methods such as hill climbing algorithms due to the high number of local optima present in many of the fitness landscapes (Mitchell, 1998). Finally, to avoid over-fitting we introduced randomness in the start position and orientation for each fitness evaluation which added noise to the fitness evaluation. These complicating factors led us to choose a search method that was sufficient, rather than hunting for an optimal search method. A comparison of the two methods we tested and our sufficiency criteria are included in the Supplementary Information S.I.-B.

#### 2.4.2 Fabrication of Branching Vine Robots

We fabricated the branching robot designs using a thermoplastic polyurethane (TPU) laser-welding technique. To adapt simulation results into manufacturable hardware, we imported the branch joint locations and angles into computer aided design (CAD) software (Solidworks) and added thickness to each line (Figure 5). With the full design outline, we exported as the file to a digitally controlled CO_2_ laser cutter. We used a laser welding approach to precisely join two sheets of TPU (Fibre Glast Stretchlon 200) along the outer seam only. Laser welding is a repeatable fabrication method for fabricating customizable pouches with a long lifespan that has previously been used to manufacture soft bending inflatable actuators (AmiriMoghadam et al., 2018). This fabrication method allowed us to simultaneously cut and seal the TPU to remove excess material from the exterior of the robot which aided the eversion process. This material was thin (which helped deployment) and elastic (which prevented creasing). The laser welded seams supported up to 70 kPa [as documented by AmiriMoghadam et al. (2018)]. Laser welding the TPU required fine tuning of the settings of the laser cutter and preparation of the TPU film with a heat-press to eliminate bubbles or wrinkles. The laser settings and TPU properties are included in Supplementary Information S.I.-D. We designed a single open end to the robot body, so mounting the robot to the rigid base only required inverting the last ∼20 cm of material and clamping that inverted part to the base. The rigid base was air-tight and contained ports for a pressure regulator. We stored the undeployed material in the base by crumpling it up instead of using a spool. This kept branches from overlapping and jamming during deployment, but meant that material is packed more inefficiently. Additionally, we observed that for successful deployment, each level of branching should be much wider than the subsequent levels. This effect may limit the total number of branches that is practical to fabricate and deploy.

## 3 Results

### 3.1 Transferability and Specialization

We expected the optimization process to generate a design that is transferable to unseen instances of a particular environment and result in specialization to the environment used during training. To test this hypothesis, we compared the performance of the designs that were each trained in different environments. Training followed the procedure described in Section 2.3 for the sensor coverage task, and was run five times with different seeds for the random number generator. The top performing designs from each environment were selected for evaluation. First both designs were evaluated on unseen instances from their own training set (i.e., the design trained on the Grid Environment was tested on its own Grid Environment training set to provide a baseline of performance). The results from these training sets show that for the same task with the same reward heuristics, the Cave Environment led to lower fitness. The two optimized designs were then each tested on unseen evaluation map sets for both environments (Figure 2E). The design trained on the Grid Environment maintained its performance on the Grid Environment evaluation set compared to the training set (within 2% fitness), and similarly the design trained on Cave Environment maintained its performance on the Cave Environment evaluation map set (within 1.5% fitness). These results show the transferability of designs trained on known maps to unseen new maps in the same environment. We also evaluated each design on the other environment (i.e., the design trained on the Grid Environment was evaluated on the Cave Environment). As seen in Figure 2E, the most-fit design for each environment was the one that was trained natively in that particular environment. This experiment showed that the optimized designs specialized to the training environment, and that the designs transferred well from training to evaluation on unseen maps in the same environment.

### 3.2 Impact of Diversity in Training Data

#### 3.2.1 Number of Known Maps

**FIGURE 3:**
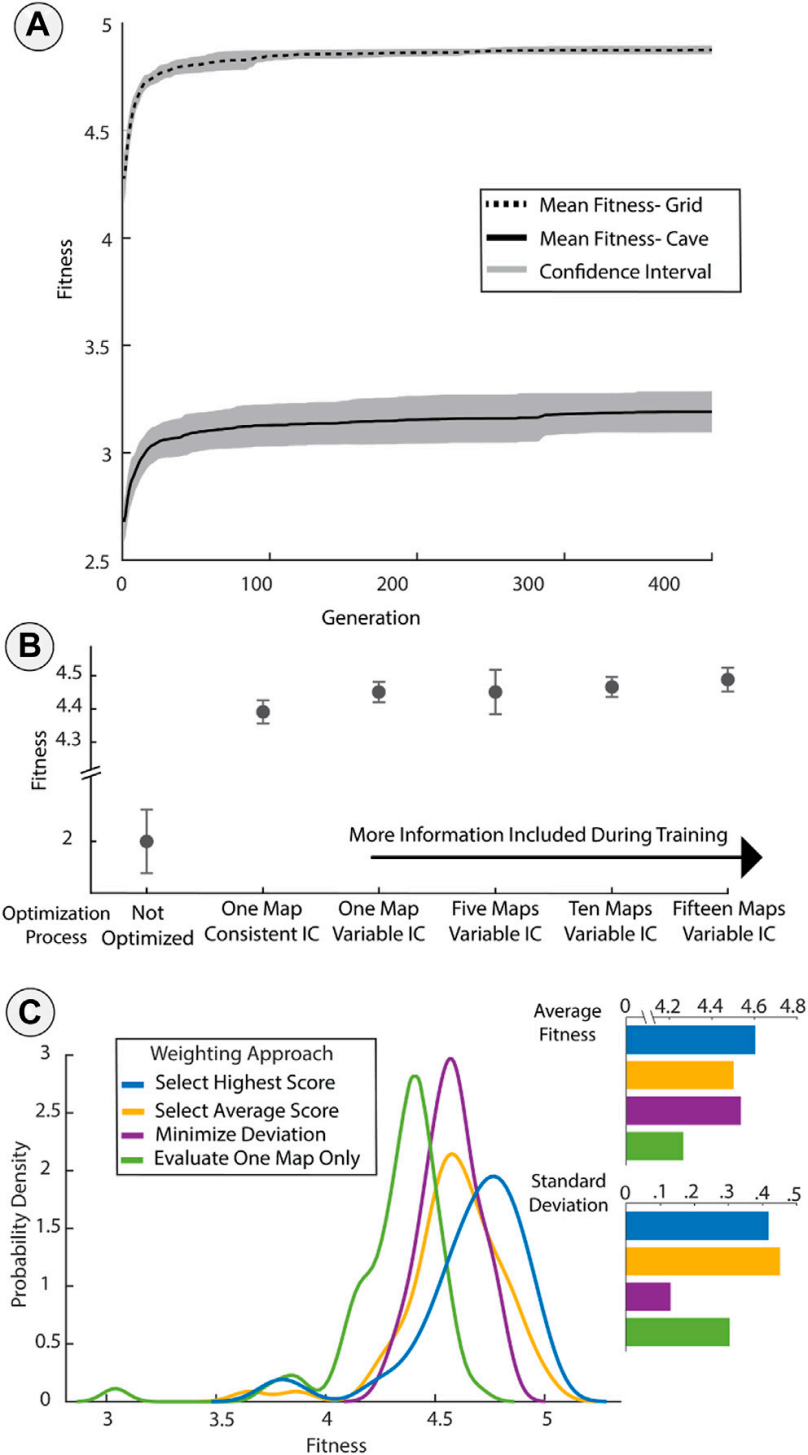
Evaluating the impact of diversity in the training data. **(A)** Plot of fitness improvement on the training maps over generational time from a sample of 10 runs. The lines represent the mean fitness and the shaded regions represent one unit of standard error. **(B)** Optimized fitness, evaluated based on unseen evaluation maps, as a function of the number and variety of test maps and robot initial conditions (IC). The baseline version of the robot that was designed heuristically (denoted “Not Optimized”) exhibited the worst performance. Using different starting positions and orientations (Variable IC’s) reduced over-fitting in the case of training based on only one known map. **(C)** Distribution of fitness scores during evaluation based on different weighting methods. Selecting the highest fitness improved performance but with a trade-off of poor performance in some cases. Minimizing deviation resulted in a consistent performance. All methods show some improvement compared to only using one fitness evaluation.

To quantify the impact of this optimization approach and to measure how much training data is required to generate high quality designs, we compared a set of designs that were optimized with different training data sets. These optimizations were run on the Grid Environment and with the sensor-coverage task. The baseline unoptimized design was manually selected using the authors’ best intuition with no further training or optimization. The optimized designs were trained on a set of known maps, and we ran different optimizations in which we varied the number of unique maps in the training set. We also tested the effect of adjusting the initial conditions (position and orientation) of the robot during optimization: the initial conditions were either constant or variable, and randomly selected- within a predefined range- from generation to generation. For the optimizations with more than one map in the training data, we measured fitness once per design per generation to make sure each design had the same number of fitness evaluations. To evaluate the performance of the designs on new maps (unseen during training), we ran the optimization five times with different random seeds with and from these five runs selected the best performing design (measured on the training set). We also measured fitness over generational time on ten runs showing the training process was repeatable (Figure 3A). To evaluate the true fitness of an optimized design, we tested the design on 25 unique, unseen instances of the Grid Environment with three different initial conditions for a total of 75 fitness evaluations.

Performance in the unseen evaluation maps was significantly improved by the optimization process when compared to the unoptimized design (Figure 3B). The unoptimized design performed poorly and resulted in high variance of fitness across different evaluation maps. The next simplest optimization, training on one map with constant initial conditions, improved performance relative to the unoptimized design but resulted in over-fitting to the single situation presented during training. Injecting noise in the training by using variable initial conditions and using additional maps both increased performance above this baseline. Adding additional maps further improved the transferability of designs, but within statistical error.

#### 3.2.2 Tuning Performance Through Weighting

The choice of weighting method had an impact on the distribution of performance of optimized designs on unseen evaluation sets (Figure 3C). During optimization, we evaluated each design on several different maps in the training set since each instance with a given initial orientation only provided an approximation of true fitness. Evaluating a design on several training maps during optimization provided more information which could lead to a better approximation of true fitness. However, the choice of how these multiple approximations were weighted and combined into a single fitness score affected the performance of the final designs. These weighting methods were a powerful design tool because they provided selection pressure and shaped the probability of different outcomes in the transfer to unseen instances. Though there were many possible weighting approaches, we selected three for evaluation: averaging, selecting the best fitness, and minimizing deviation. In the optimization that minimized deviation, 25% of the composite score was based on the average fitness to ensure there was some pressure to improve performance. We optimized each design on the Grid Environment for the sensor coverage task, varying only the weighting method used. We also compared the results to an optimization run with no weighting (with only one fitness evaluation per design). We then tested these designs on unseen evaluation maps from the same Grid Environment and compared the distribution of the resulting fitness scores. Figure 3C shows the probability distribution for the scores of each design in the evaluation set. Selecting the highest score resulted in “greedy” performance, with the best possible outcomes during evaluation but with a higher spread in the distribution including some poor scores. Averaging scores surprisingly had a lower average and also a wider deviation of performance than the multi-objective weighting that also minimized the standard deviation. A weighting that only considered the deviation would not necessarily select for high performance but combining the mean and standard deviation into a single score resulted in a design that showed both high performance and consistency across the set of evaluation maps. The weighting method selected had a noticeable impact on the distribution of performance on unseen maps, but came at the cost of increased computational effort through additional fitness evaluations in each generation.

### 3.3 Unique Capabilities of Branching Vine Structures

#### 3.3.1 Reachability and Coverage

**FIGURE 4:**
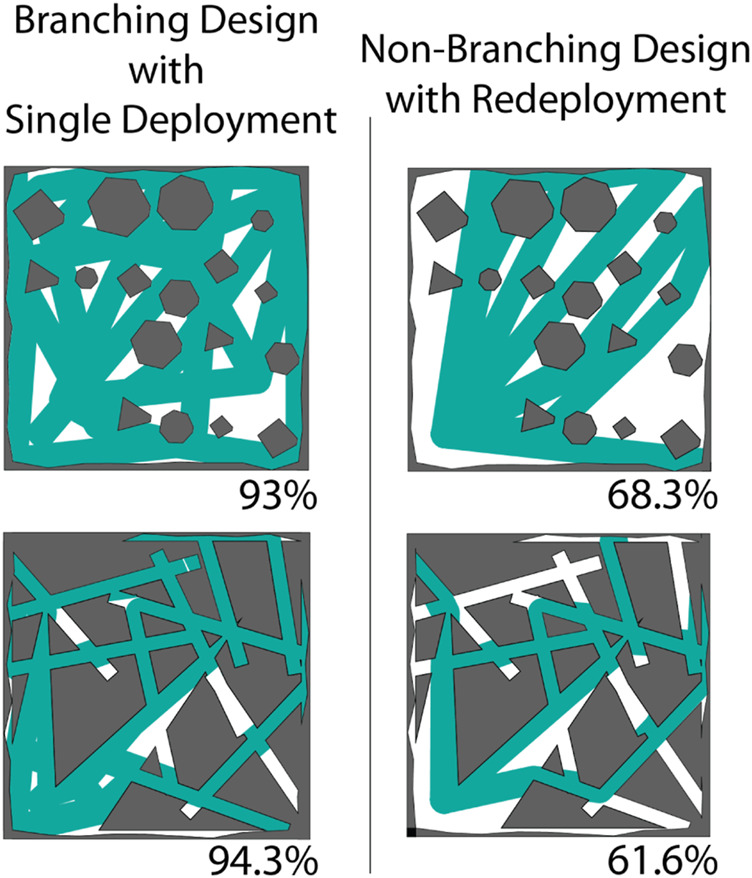
Comparison of the reachability of the space. Reachability of a single branched deployment in the Grid and Cave environments (Left). Union of the reachability of a straight tube robot over many redeployments in both Grid and Cave Environments (Right). Even allowing many redeployments, a vine robot without branching and without active steering cannot access large areas of the space. In contrast an optimized branching robot can cover much more of the space once deployed, also without active steering. Branching successfully mitigates the issue of trajectory condensation and provides other interesting properties such as covering multiple locations simultaneously and distributing forces.

To compare branched and single-path designs, we studied the reachability of each in both of the previously mentioned types of simulated environments. As previously reported, eversion robots running open-loop are limited by trajectory condensation through interaction with obstacles (Greer et al., 2020). Branched designs were able to recover non-redundant mobility through a space by deploying new material that was independent of prior obstacle interactions (Figure 4). To compare these two classes of designs and explore the issue of trajectory condensation, we used a set of simulations to compare the reachability of these design classes. For the single-path robots, we ran 50 sequential deployments, each with a different initial orientation. In an example from the Grid Environment, these 50 unique initial configurations were reduced into six final configurations. Similarly, in the Cave Environment, the 50 unique initial configurations only resulted in five final configurations once growth was complete. For each of these instances we considered a buffer around the path of the robot to be a part of it is reachability, then took the union of this area across each of the 50 deployments. For the branched systems, we optimized the design for each environment with the reward heuristic that measured a buffered reachable area surrounding the path of the robot. The reachability of the branched design was only calculated from a single deployment. On the Grid Environment, the branched design reached 93.0% of the space with a single deployment compared to the multiple deployments of the non-branched design which covered 68.3% of the space. In the Cave Environment, the branched design also outperformed, covering 94.3% of the space compared to the non-branched which reached 61.6% of the space (Figure 4).

#### 3.3.2 Demonstration of Deployment for Sensor Coverage

**FIGURE 5:**
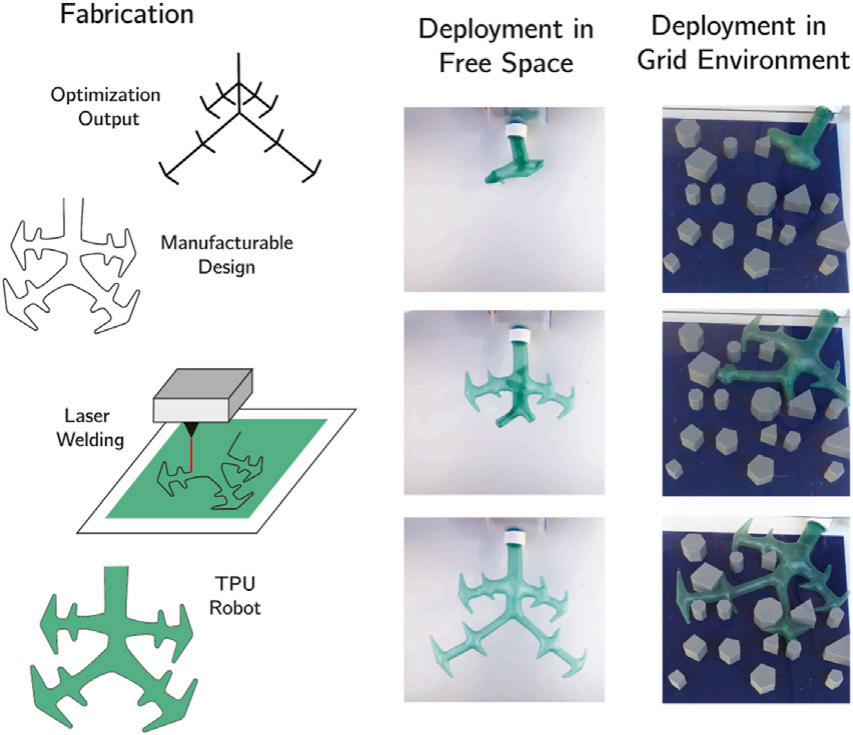
Demonstration of fabrication and deployment of a branching vine robot. To manufacture the designs we added thickness to the line-output from the optimization process, and cut the robot out of TPU using a laser-weld process (see Supplementary Information S.I.-D). Decreasing the diameter of the robot at each hierarchical branch-level improved deployment. We also tested deploying the branching vine robot both in free space and the grid environment.

We demonstrated the fabrication and deployment of a design based on the output of the optimization to explore how these results may be translated into hardware. We created a manufacturable design from the output of the optimization by adding thickness to each branch and fillets to each joint, where size was scaled by the level of branching hierarchy (Figure 5). We evaluated the branching robot on an example map fabricated with 3D printed obstacles. We used a laser welding fabrication approach to ensure precision and repeatability in the manufacturing process. This approach simultaneously cut and sealed layers of TPU and was previously used to make soft bending actuators (AmiriMoghadam et al., 2018). To deploy the system, we took the cut TPU and placed it inside a pressure vessel, inverting and attaching the non-branching end. Our approach was repeatable and allowed for redeployment after manually resetting the system. Through hardware validation we observed several properties. Though the device was scalable in overall size, deployment was easier at lager sizes since there was less internal friction to overcome while transporting new material to the tip of each branch. The device was reusable, but required manually resetting the robot between trials, and over time the TPU seal developed leaks. Furthermore, head-on collisions with obstacles, especially at branching vertices, sometimes prevented growth [though it may be possible to overcome this effect with nutation (Wooten and Walker, 2018; Taylor et al., 2021)]. Though the path taken by the robot was highly sensitive to its initial position and orientation, the optimized design was often able to navigate through the environment due to its branching structure and high degree of compliance.

#### 3.3.3 High Strength Anchor Demo

**FIGURE 6:**
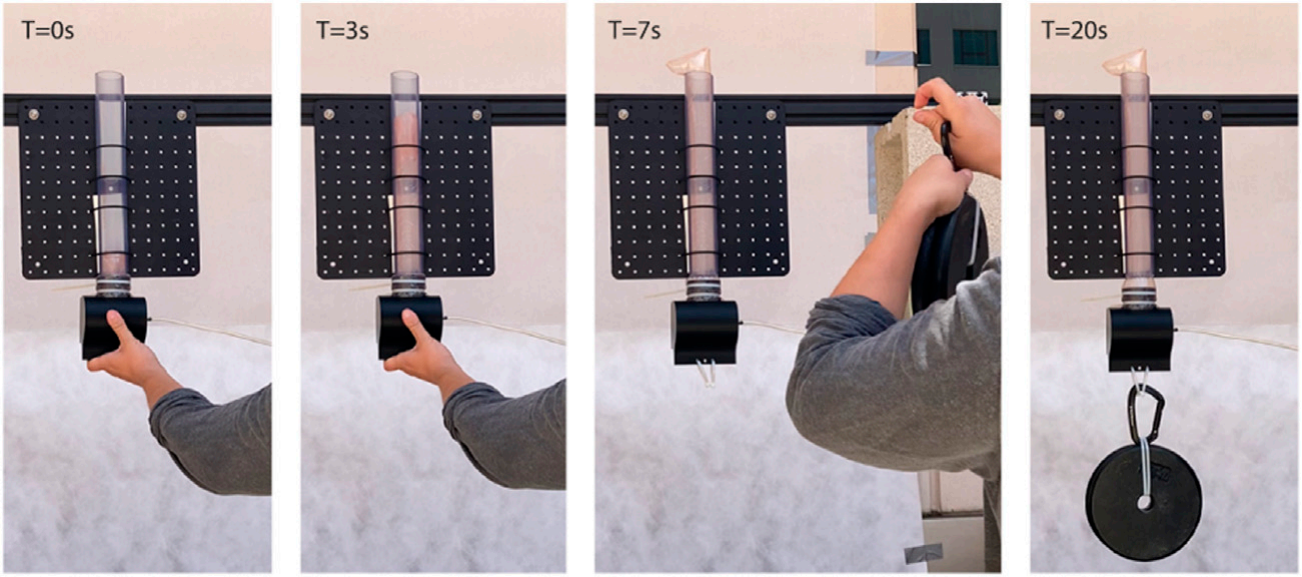
Self-deployment with anchoring. We manually placed the robot at the entrance of a clear pipe (0 s) and subsequently applied 100 kPa of pressure to deploy the system. Once fully deployed (7 s), we added 66.7 N while maintaining the pressure. The robot held its position until pressure was released.

To hold the payload seen in Figure 1, we adjusted our materials and fabrication methods to support greater force. We used a thicker TPU (0.05 mm compared to 0.0375 mm) (Stretchlon 800, Fiberglast) which prevented the body of the robot from significantly deforming or tearing under load. This thicker TPU prevented a quality laser-weld seam which was our method for creating a branching design. To accommodate this change we used an impulse sealer to create a straight tube without branching. With the non-branching design, we positioned the robot below a target passageway, deployed the robot through the passageway, and then suspended a payload to the base of the robot. The robot held 66.7 N (15 lbs) at an internal pressure of 100 kPa, the same pressure used to deploy the robot through the passageway (Figure 6). This approach shows an interesting approach to supporting high forces with soft materials, but relying on growth instead of grasps (Glick et al., 2018). The maximum payload was determined by the mechanical connection of the robot to the base, preventing comparing model predictions to payload capacity.

#### 3.3.4 Optimized Anchoring Forces With Hardware Test

**FIGURE 7:**
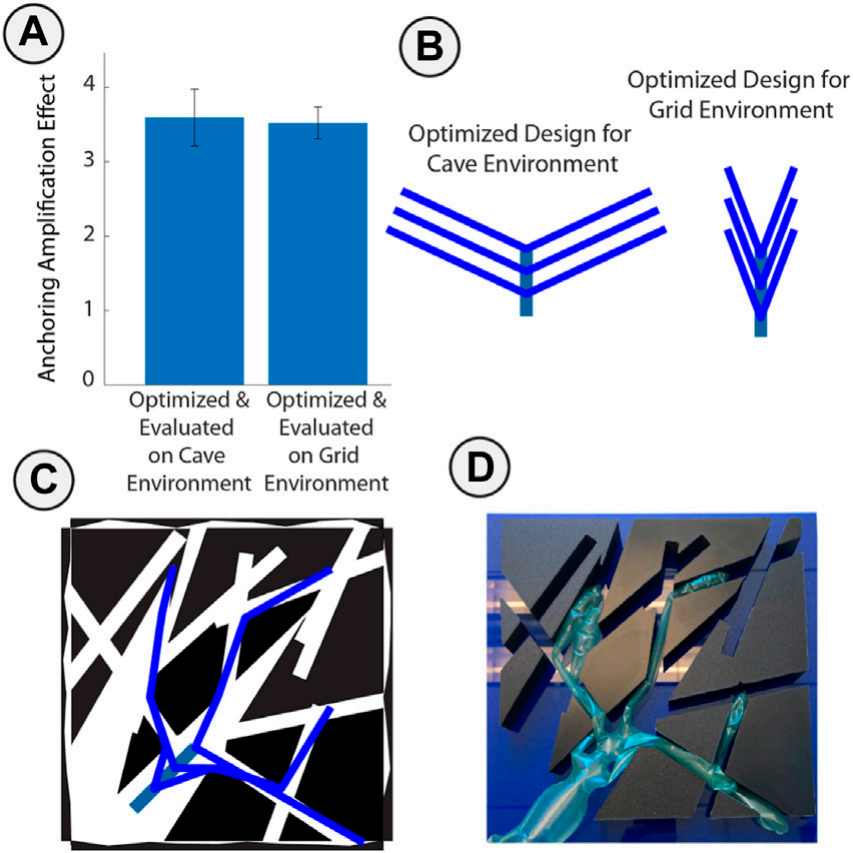
Optimization and hardware validation of anchoring. **(A)** Anchoring amplification effect of an optimized branching vine robot compared to a single pouch anchor in both cave and grid environments. **(B)** Optimization output of similar designs from different training environments. **(C)** Simulated final position of optimized design in cave environment. **(D)** Manually deployed robot to ensure alignment with simulation, used to compare to the anchoring force predicted by simulation.

Branching provided a significant opportunity to grip inside of an environment due to capstan friction and the pouch-anchoring effect. We optimized two designs in simulation according to the task described in section 2.2.2 on the Grid and Cave environments (Figure 7). We simplified the design space and added a pouch to the end of each branch to improve the accuracy of our anchoring force prediction by controlling the contact location with the wall. The similar force capacity and optimized design between the designs optimized in the cave and grid environments show this problem has a simpler solution compared to other tasks, especially since we do not penalize for length or design complexity in this task. We also fabricated an example Cave Environment using 3D printed obstacles to validate the force capacity. We measured the anchoring force by manually deploying the robot in the environment then measuring peak pull-out force with a digital force gauge (Mark 10). The robot was manually deployed to ensure consistent placement between trials and to accommodate for challenges with growth at this small aspect ratio. For this experiment we tried to enforce anchoring only at the tip so shrunk the branch width. This prevented repeatable growth. A single pouch with no capstan effect held 0.65 N, and the optimized design held 4.25 N when manually deployed. The measured anchoring magnification was 6.54, compared to the model prediction of 6.86. This difference comes from the assumption in the model that each branch makes good contact and evenly shares the load. In the experiment, we found that out of the six branches, one did not make contact with the sidewalls and another had slack preventing it from sharing the load. This method of anchoring does not rely on a branching design, indeed, the capstan effect grows exponentially along the length of a single branch with respect to the total change in angle. However, each added branch adds to the total anchoring force and more importantly, adds redundancy in case any single branch cannot make good contact with the cave walls. The approach of deploying into an environment and subsequently loading the robot is an interesting alternative to traditional robotic gripping and manipulation. Furthermore, this test showed the adaptability of the optimization approach to scale to a variety of tasks.

## 4 Discussion

In this work we showed that the combination of branching vine robots and task-specific optimization enable a new approach to navigating unmapped environments. This optimization framework supports specialization of designs to a range of tasks and environments. Furthermore, we found that branching improved reachability within a cluttered space, promoted simultaneous coverage of a large area, and enabled anchoring—mirroring the properties found in biological growth. We used a versatile and precise fabrication approach for the manufacture of the optimized designs. Though capstan friction has been studied as a limiting factor for vine robots, we show that it can be harnessed to amplify the pouch anchoring effect. This effect has the potential to enable new kinds of grippers and anchors in cluttered environments. Though deployment and manufacturability were limited by practical constraints such as internal friction, the pressure-rating of the TPU seam, and head-on collisions, it may be possible to mitigate these limitations by including appropriate fitness penalties during optimization. Additionally, though this work only considered two-dimensional designs, future work could explore the application of these concepts to three-dimensional space. However, the benefits of branching vine robots do come at the cost of increased complexity. Both the fabrication and deployment of branching designs require further study for large (i.e., > 2 m) and small (i.e., < 0.25 m) scales. Additionally, approaches to adding sensors along the hierarchical body and communicating within the structure are open challenges. Finally, for deployment in a known environment, an optimized open-loop or a closed-loop growing robot will likely be able to match performance for most tasks with less design effort.

## Data Availability

The raw data supporting the conclusion of this article will be made available by the authors, without undue reservation.
