# Supplementary material for: Branching Vine Robots for Unmapped Environments
Source: Front Robot AI. 2022 Mar 24;9:838913. doi: 10.3389/frobt.2022.838913 (PMC8987124; doi:10.3389/frobt.2022.838913)
Supplement: Supplementary file 1 [file DataSheet1.pdf]

I. Supplemental Information

A. Computer Generated Environments

In this work we used two computer generated environments: a Grid Environment and a Cave Environment. To allow for fair comparison between designs optimized for each environments they have the same size and are enclosed spaces. A 100×100 unit box bounded every map in both environments. The outer walls had a randomized texture to prevent the design search from over-exploiting growth along these outer boundaries. Additionally, both environments had an 15×15 empty region in the bottom left of the space to allow for randomized initial conditions at each fitness evaluation. The Grid Environment was comprised of a grid of convex polygons. The randomized variables were: i) the number of polygons in a particular row, ii) the number of rows, iii) the number of points in a particular polygon, iv) the size of a particular polygon, and vi) the orientation of a particular polygon. Maps in the Cave Environment began with a solid 100×100 obstacle and subsequently had randomized pathways cut out from the initial solid obstacle. These pathways were rectangles with dimension 70 units by 5 units. Three of these pathways were initialized at 4 distinct locations ([25,60],[75,25],[80,80],[55,55]) for a total of 12 pathways subtracted from the initial obstacle. We then added a random rotation and added/subtracted a random value to the X & Y coordinates of the centroid of each obstacle. Finally, two channels with randomized tip locations are added to connect the starting region to the network of pathways.

B. *Search Performance*

Below (Fig. S.1.) is the data used to generate the results in Fig. 3-A. We performed 10 runs to 400 generations each. We ran each experiment in both the Cave and Grid type environments. The small variance and quick climb of fitness in the Grid environment suggests that it was a smoother fitness landscape than the Cave environment. We also show the output of the best performing individual from generations 1, 10, 100, and 400 from one run of optimization in Fig. S.2. This is a single, representative example to illustrate the overall improvements in efficiency and coverage of the area.

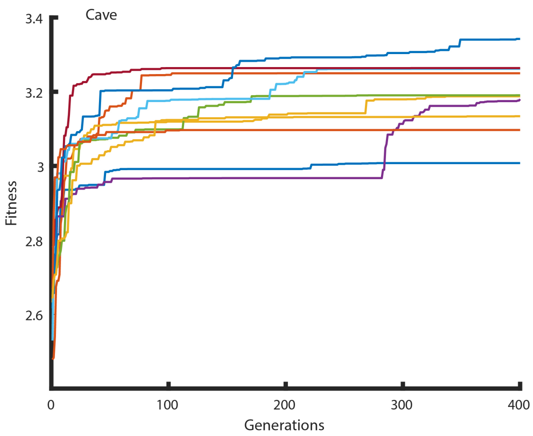

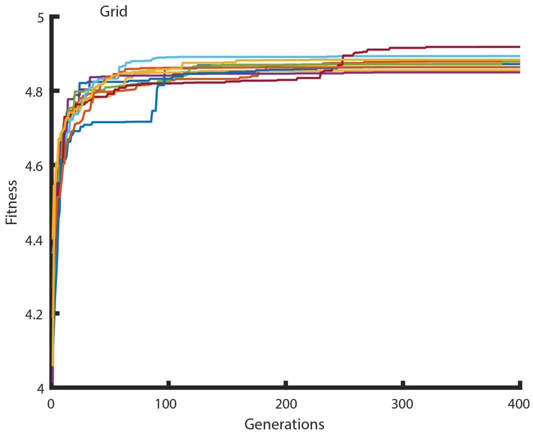

Fig. S.I.1. Raw fitness output from optimization.

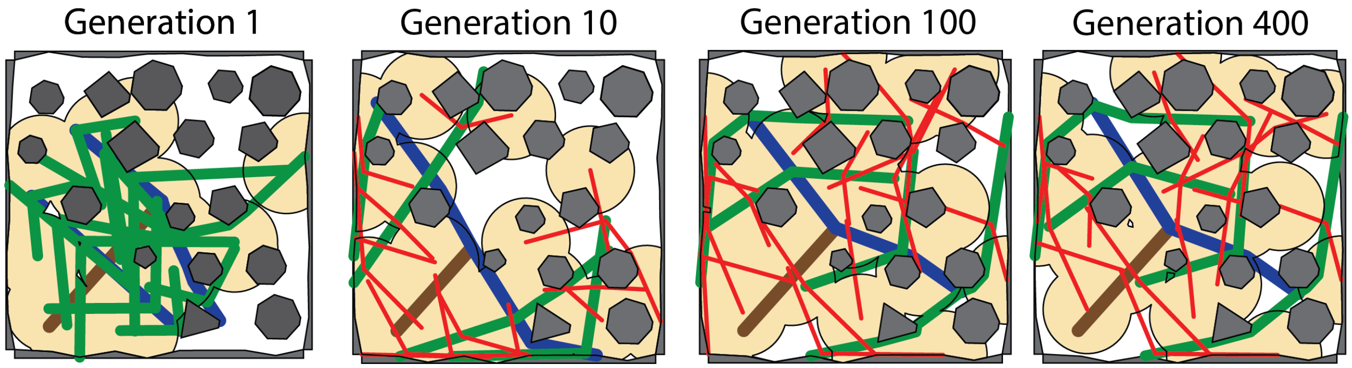

Fig. S.I.2. Visualization of representative design improvement during training. These plots are only illustrative since design performance was evaluated on multiple maps with different starting locations. The increase in total area covered and efficiency of the length of the design show the progress during optimization.

C. Search Method Comparison

To verify that a given search method was finding quality solutions, we used a simplified task that did not rely on obstacle interactions or introduce randomness into the fitness evaluation. For this task, we initialized the given design in an open space with no obstacles present. We rewarded the unique area covered and penalized the total length of the design ($fitness = 6 * area - covered - 0.02 * total - length$). To improve understanding of the fitness, we set the penalty for length to be an order of magnitude smaller than the area for reward. This meant that an optimal design should have maximized the number of sensors with no overlap between the sensed areas. Any designs that met these criteria could then be compared through the total length used to maximize this sensor area (where a shorter length is better). While analogous to a circle packing problem, the parameterized design of the robot added additional constraints which made the optimized solutions less dense. A comparison of the results from a genetic algorithm and particle swarm algorithm are seen in Fig. S.2. The resulting design shown in (Fig. S.3-A) was generated by the custom evolutionary algorithm and those shown in (Fig. S.3-B) was generated by a commercially available particle swarm search (Matlab). The total length of the design in Fig. S.3-A was 2303.1, and the total length of the design in Fig. S.3-B was 2303, a difference in length of .005%. To test the reliability of the results, we ran five trials of the evolutionary algorithm, and only two of the five solutions were within 5% of the optimal length found by the particle swarm search. The fact that three of the five random seeds produced sub-optimal designs demonstrates the risk of getting stuck in a local optimum. Finally, the two optimized designs in Fig. S.2 have nearly the same fitness but with distinct morphologies, showing that there may be multiple fit design solutions for a certain task with this type of robot. For the evolutionary algorithm we used a (μ+λ) method with an elite pool of six in a population of 54 to preserve promising candidates since each fitness evaluation only provides an approximate estimate of true fitness.

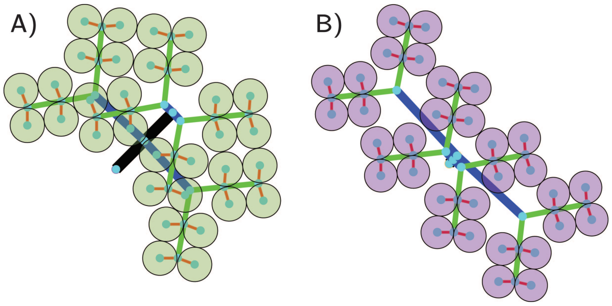

Fig. S.I.3. Comparison of the results with the two different optimization approaches. Though the morphologies generated by a custom evolutionary algorithm (A), and a commercially available particle swarm search (B) were qualitatively different, the fitnesses of the two designs were within 0.005%..

Sensor Coverage Heuristic

Area Factor (AF) was based on measured area covered as a percentage of the total possible area. Area Efficiency (AE) and Length Efficiency (LE) were intended to penalize overlapping area i.e. promote unique coverage and were split up to allow for different costs based on design or fabrication constraints. Length Penalty (LP) was used based on total length of the design to promote shorter designs where coverage or efficiency were equal. Each of these variables had a scalar of +1 added which was a legacy from older heuristic tests.

$AF = (AreaCovered/22582)$

$PA = NumberOfSensors * \pi * 15^2$

$AE = (PA - AreaCovered)/PA$

$LE = AE * (TotalLength/NumberOfSensors)/65$

$LP = TotalLength/37440$

$Reward = -10 + 6.5AF - 1.75AE - 0.5LE - 0.25LP$

Anchoring Heuristic

$Reward = \sum_{i=1}^{n} F_{Anchor}$

D. *Laser Settings and TPU properties*

We used a laser welding process for repeatable fabrication of the branching vine robots. This fabrication method allowed us to simultaneously cut and seal the actuator to remove excess material from the contractile section of the actuator which can restrict motion. We used a thermoplastic polyurethane (Stretchlon, Fibreglast) which can stretch up to 400-500% its original length. We performed tensile tests on five samples to test the elasticity and strain of the TPU material. The samples were strained at 0.05 mm/mm/s until failure. We ultimately did not use this relationship in our models, but have provided it as a reference.

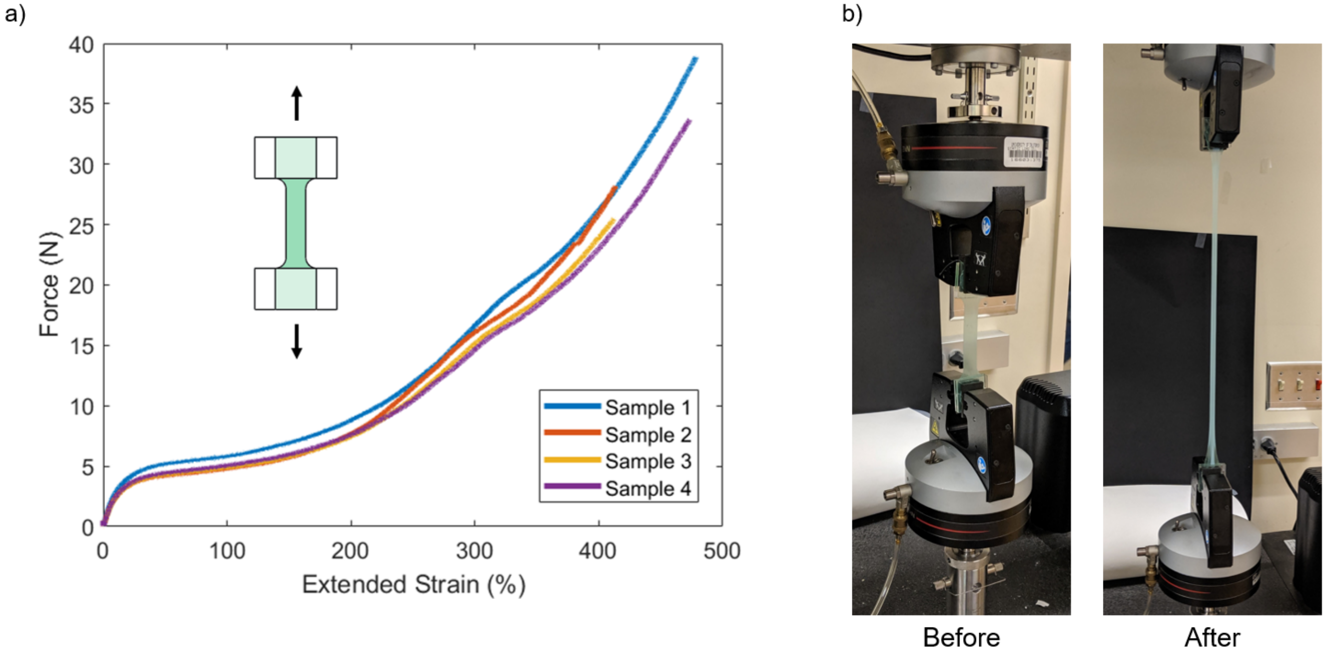

S.I.4. a) Tensile testing of the TPU material. b) Stretching TPU material 400%-500%.

This material was robust, making it a good material choice for a collapsible robot. Laser welding the TPU required fine tuning of the laser cutter settings until the pouch successfully sealed. To laser weld the TPU layers, we used the following procedure:

Steps for TPU Laser Sealing

1) Place the TPU layers on heat press and roll out any bubbles.
2) Press both layers of tpu at 77°F for 2 minutes
3) Let the TPU cool on the heat press for 5 minutes

To laser weld the actuator, we used a commercial, digitally controlled $C0_2$ laser machining system (PLS6MW, Universal Laser Systems). We used two sets of settings to cut and seal the actuator. The first set of settings cut and sealed the the actuator simultaneously. Those settings were 80 % power, 100 % speed, and 500 pulses per inch at a bed height at 0 mm. We also tested settings that just sealed the top TPU layer to the bottom layer. These settings were 20 % power, 100 % speed, and 500 pulses per inch at a bed height at 0 mm.

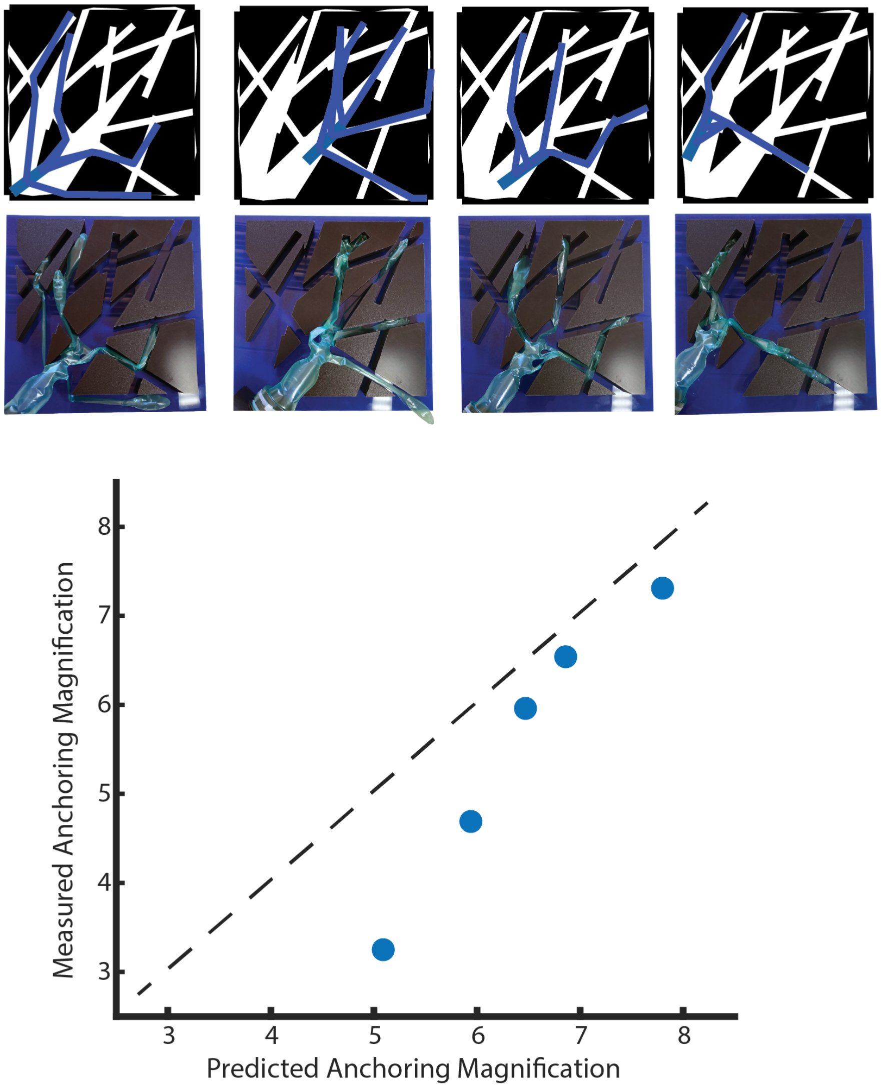

S.I.5. Repeatability of anchoring. Top) different configurations tested against model predictions. Bottom) Predicted and measured force of the different configurations. 1:1 Dashed line added for reference to compare prediction to measured. Model predictions seem to better match in configurations where anchoring is more effective.

E. Anchoring Tests

We evaluated how consistent the anchoring effect was by comparing several simulated predictions to measured forces in the same configuration. The robot was manually placed in the configuration due to the aspect ratio and internal friction of the branches. We used the same map and same robot design for every trial. Results showed that the accuracy of the prediction depended on configuration of the robot. Qualitatively, we observed that redundant branches in the same passageway seemed to not contribute well to overall anchoring force and may have caused of error in the prediction. Indeed in these cases, sometimes individual branches twisted and were unable to even inflate.
